# Safety Analysis of *Panax Ginseng* in Randomized Clinical Trials: A Systematic Review

**DOI:** 10.3390/medicines2020106

**Published:** 2015-06-08

**Authors:** Young-Sook Kim, Jung-Yoon Woo, Chang-Kyun Han, Il-Moo Chang

**Affiliations:** The Korea Ginseng Research Institute, Korea Ginseng Corporation, Daejeon 305-805, Korea; E-Mails: yskim@kgc.co.kr (Y.-S.K.); 20130021@kgc.co.kr (J.-Y.W.); ckhan@kgc.co.kr (C.-K.H)

**Keywords:** *Panax ginseng*, randomized controlled clinical trial, safety

## Abstract

**Background:** *Panax ginseng* C.A. Meyer is one of the most frequently used herbs in the world. The roots of *Panax ginseng* have been used as a traditional tonic and medicine for thousands of years in Korea and China. Today, ginseng root is used as a dietary supplement and complementary medicine and for adjuvant therapeutics worldwide. The efficacy of ginseng has been studied in a wide range of basic research and clinical studies. However, it has been reported that the results from clinical studies are conflicting, and they depend on the parameters of the protocol design including the conditions of the participants and the types of ginseng used such as red ginseng, white ginseng, fermented ginseng and cultured ginseng. Meanwhile, in addition to clinical efficacy, the safety of ginseng is a highly important matter for customers. With globally increasing demand for *Panax ginseng* as a dietary supplement or complementary medicine, it is necessary to provide information on its safe use to customers to improve their health conditions. Although the safety of *Panax ginseng* in pre-clinical studies is well known, the evaluation of safety in clinical studies has so far been insufficient. This systematic review was conducted to assess the safety of ginseng in randomized controlled clinical trials (RCT) over the last 10 years. We chose the last 10 years because many clinical trials have been conducted in the past 10 years, and it will help to understand the recent trends in RCTs of ginseng.

**Methods:** Articles on ginseng studies were searched with keywords in MEDLINE and four other Korean online database sites. Studies with ginseng as a monopreparation were selected while studies with single administration, preparations combined with other herbs or drug combinations were excluded from the selected studies. Data from the selected studies meeting the criteria were extracted and reviewed in terms of study design, condition and number of participants, type of ginseng, dosage, duration, main results, adverse events and adverse reactions.

**Results:** Forty-four studies met the selection criteria. These studies covered the efficacy of ginseng in areas such as cardiovascular function, glucose metabolism, sexual function, anti-oxidation, anti-fatigue and psychomotor function. Twenty-nine studies showed positive results while fifteen studies showed no effect. Sixteen studies reported adverse events while five studies had no adverse events. Twenty-three studies did not mention any adverse events. The main adverse events of ginseng reported were general symptoms such as hot flushes, insomnia and dyspepsia with no significant difference in frequency and symptoms between the ginseng and placebo groups. The symptoms were mild and temporary with no serious or severe adverse events.

**Conclusion:** *Panax ginseng* showed a very safe profile in a limited number of RCTs with a small number of participants with various conditions ranging from healthy participants to patients with symptoms. However, to increase the usefulness and lower the health risk of *Panax ginseng* to customers, clinical trials on a larger scale and with a higher standard are necessary to define its efficacy and safety as a dietary supplement or complementary medicine.

## 1. Introduction

*Panax ginseng* has been used not only as a medicine but also as a restorative and prophylactic remedy for thousands of years in Asia. Ginseng is classified as fresh ginseng (raw ginseng), white ginseng (dried after peeling) and red ginseng (steamed and dried) depending on how it is processed. Ginsenosides are the most studied active components in ginseng. About 40 types of ginsenosides are contained in ginseng along with non-saponin compounds like acidic polysaccharides and polyacetylenes [1,2,3]. Much basic research on the range of efficacies of ginseng, including its immune-enhancing, anti-fatigue, and anti-cancer functions and improvements to cardiovascular function, is ongoing, along with numerous studies on its mechanisms [4]. 

Currently, the demand for ginseng as a functional food has been increasing not only in Asian countries like Korea, China and Japan but also in Western countries. Despite this trend, RCTs evaluating the efficacy of ginseng as a functional food and as an evidence-based complementary medicine are still very limited. Therefore, it is necessary to evaluate the efficacy and safety of ginseng along with the increased consumption of ginseng as a functional food, adjuvant or complementary medicine. A number of clinical studies on the efficacy and safety of ginseng in randomized controlled clinical trials have been reported [5,6]. The object of this systematic review is to summarize the randomized clinical studies of the past 10 years, focusing on the safety of ginseng to promote its usefulness as a functional food or complementary medicine.

## 2. Methods

### 2.1. Data Sources and Selection

A systematic literature search was conducted on MEDLINE, Korean Studies Information Services System (KISS), NuriMedia database (DBPIA), Korea Institute of Science and Technology Information (KISTI) and the literature search system of the authors’ own institute. Keywords used in the searches were “*Panax ginseng*” or “clinical” or “randomized” or “controlled” or “human study”. The search period ranged from January 2005 to November 2014. Further publications until December 2014 were also searched. Two independent reviewers (JW and YK) assessed all titles and abstracts with a pre-defined inclusion criteria using the following united keywords: *Panax ginseng*, ginseng, controlled, trial.

### 2.2. Eligibility of Studies

Studies were selected in accordance with the following criteria: (1) randomized controlled clinical trial; (2) *Panax ginseng* as a monopreparation as the intervention; (3) subjects with no other medication or supplement intake; (4) single administration excluded; (5) studies using other parts of ginseng besides the root excluded; (6) administration routes other than oral intake such as intravenous or dermal application excluded; and (7) articles in languages other than English or Korean excluded.

### 2.3. Data Extraction

The data were extracted from the articles according to the predefined criteria: study design, condition and number of participants, type of ginseng, dosage, duration, main results and adverse events.

## 3. Results

### 3.1. Included Studies

**Figure 1:**
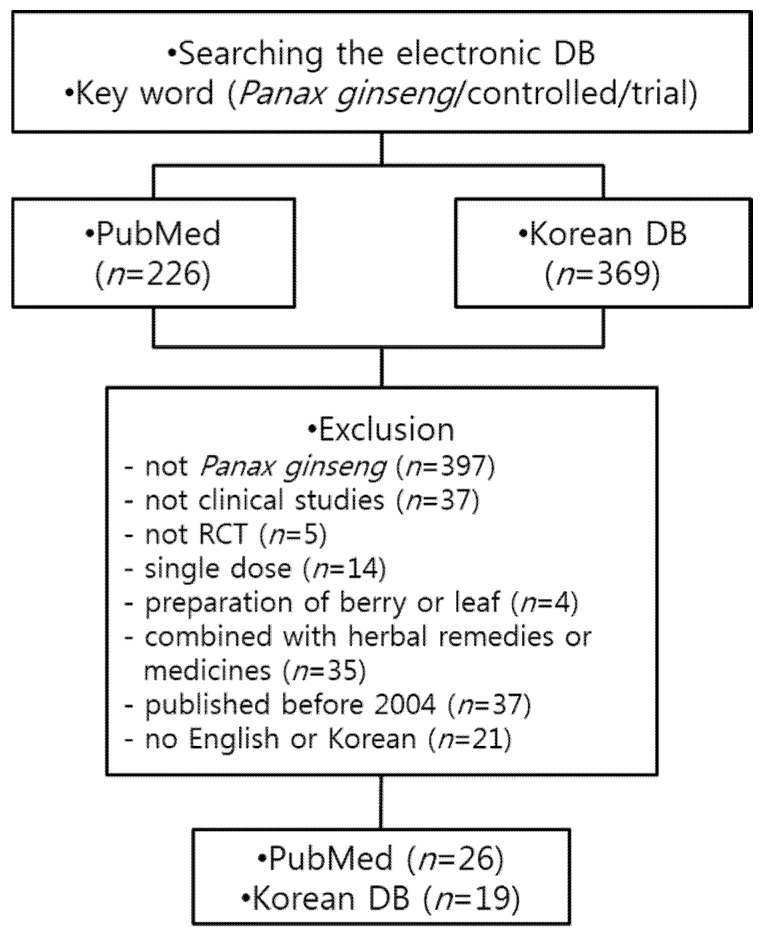
Flow diagram of the publication selection process. RCT: Randomized controlled clinical trials.

Out of 595 articles that were initially chosen from MEDLINE and four other Korean DB sites using the keywords, 45 articles met all the criteria (Figure 1). To put more emphasis on safety rather than efficacy, studies that used ginseng with other herbal medicines or took other drugs together were excluded from the review because ginseng and other herbal medicines or drugs could cause interactions resulting in adverse events. The 45 articles meeting the criteria of this study are summarized in Table 1.

Because two of the 45 articles were from the same clinical trial study [7,8], 44 studies met all the criteria for the time period from 2005 to 2014. One study was single-blind [9], and the rest were double-blind studies. There were 7 studies with crossover design while the rest were parallel design studies. The areas of study for the included studies were as follow: sexual function (6 studies), glucose metabolism (5 studies), cardiovascular function (4 studies), psychomotor function (3 studies), fatigue (4 studies), antioxidant function (3 studies), obesity (3 studies), sleep (3 studies), menopausal symptoms (2 studies), cancer (1 study), respiratory system (1 study), hearing (1 study), fibromyalgia (1 study), safety and tolerability (1 study), dry mouth (1 study), somatic symptoms (1 studies), androgenic alopecia (1 study), general symptoms (1 study), gynecological complains (1 study), and depression (1 study).

### 3.2. Participants

In total, 3092 participants were involved in the 44 selected studies. The median number of participants was 53 (range: 15–643). Participants were aged between 18 and 79 years. Among the 22 studies conducted on healthy subjects, 9 studies involved only male subjects, 2 studies involved only female subjects, and 11 studies included both genders. There were 6 studies on menopausal females, 5 studies on obesity, diabetes or metabolic disorders, 4 studies on erectile dysfunction, and one study each on chronic gastritis, chronic fatigue, female sexual dysfunction, chronic rhinitis, fibromyalgia, dry mouth, and alopecia, respectively. There was one study on healthy Koreans and Chinese for general symptoms and adverse events.

### 3.3. Interventions 

Out of 29 recent studies on Korean red ginseng, 23 studies used the powder type, 5 used the extract type and 1 used a ginsenoside-fortified ginseng extract. Four studies used *Panax ginseng* extract (G115), 1 study used powder, 3 studies used 20% ethanol extract, 5 studies used fermented red ginseng powder and 2 studies used cultured ginseng.

**Table 1:** Summary of randomized controlled studies of *Panax ginseng* for a 10 year period (2005–2014).

Author (Year)	Design	Condition, Age Range (yr)	No of Subjects C/T	Intervention Dose (g/day) Duration	Main Results	Adverse Events	Adverse Reactions
Kang (2013) [10]	DB, parallel	Healthy subjects, 30–50	C and T:20	Korean red ginseng powder, 1.5 g, 8 weeks	1. Temperature differences of specific part: NS Maximum/average rate of blood circulation: NS Blood coagulation/lipids: NS	Not reported	-
Park (2012) [11]	DB, parallel	Subjects with metabolic syndrome ≥ 20	C:25 T:23	Korean red ginseng powder, 5 g, 12 weeks	1. Blood pressure: NS Glucose, lipids: NS CRP ; NS	C:1 T:0	Gastrointestinal disturbance (C:1)
Choi (2009) [12]	DB, crossover	Healthy, married women with FSFI score below 25, 30–45	C and T:23	Korean red ginseng powder, 3 g, 6 weeks	1. Thermal effect measured with subjective warm scale and DITI: NS	Not reported	-
Shin (2007) [13]	DB, parallel	Healthy subjects with cholesterol 180– 250 mg/dL, 20–59	C:29 T1 (low-dose): 29 T2 (high-dose): 29	Korean red ginseng extract, 1.5 g, 3 g, 8 weeks	1. Inhibited platelet aggregation PT, APPT: NS	T:1 (not clear in dosage)	Cold allergy (T:1)
Bang (2014) [14]	DB, parallel	Subjects with IFG (100–125 mg/dL), IGT (2-h OGTT ≥ 140 mg/dL) or newly diagnosed T2DM, 20–70	C:20 T:21	Korean red ginseng powder, 5.0 g, 12 weeks	1. Decrease in insulin and C-peptide level at 30 min during OGTT	Not reported	-
Oh (2014) [15]	DB, parallel	Healthy subjects (FBG5.6–7.8 mmol/L), 44–62	C:21 T:21	Fermented red ginseng, 2.7 g, 4 weeks	1. Reduction in postprandial glucose level and glucose AUC Increase in postprandial insulin levels	C:0 T:1 (exclude the data)	Hypoglycemia (T:1)
Lee (2013) [16]	DB, parallel	Post-menopausal women, 52–64	C:44 T:49	Fermented red ginseng, 2.1 g, 2 weeks	1. Decreased HbA1C, insulin and HOMA-IR 2. Increased DHEAS, GH and E2	Not reported	-
Reed (2011) [17]	DB, parallel	Overweight and/or obese subjects (BMI 34 ± 1 kg/m^2^) with impaired glucose tolerance or newly diagnosed T2DM 43–49	C:5 T1:5 T2:5	T1: Korean red ginseng extract, 3 g/day for 2 weeks→8 g/day for 2 weeks T2: Re, 250 mg/day for 2 weeks→500 mg/day for 2 weeks	1. OGTT, β-cell function, or multiorgan insulin sensitivity: NS	Not reported	-
Reay (2009) [18]	DB, crossover	Healthy subjects, Study 1: 33.4 ± 10.4 Study 2: 38.4 ± 10.6	Study 1:C and T 23 Study 2:C and T 14	Study 1: *Panax ginseng* extract (G115), 200mg, 8 weeks Study 2: Korean red ginseng extract, 200 mg, 8 weeks	1. HbA1c: NS Plasma insulin:NS	Not reported	-
Yeo (2012) [19]	DB, parallel	Healthy young men, 19–25	C:7 T:8	Korean red ginseng 4.5 g, 2 weeks	1. Neurocognitive function test (Vienna test system version IX): NS	Not reported	-
Reay (2010) [20]	DB, crossover	Healthy, young volunteers, 18–26	C and T:30	*Panax ginseng* extract (G115) 200 mg, 8 days	1. Working memory: NS Mood: NS	Not reported	-
Kennedy (2007) [21]	DB, crossover	Healthy, young volunteers, 19–25	C and T:18	Korean red ginseng extract, 200 mg, 8 weeks	1. Improved working memory and mood and quality of life Blood glucose, insulin, HbA1c: NS	Not reported	-
Yun (2010) [22]	DB, parallel 3 years of intervention and 8 years of follow up	Chronic atrophic gastritis patients, 40–69 (no mentioned medication)	C:318 T:325	Korean red ginseng extract powder, 1 g/week, 3 years	1. Cancer case occurred, C:16, T: 8 2. Cancer risk in T included both gender: 0.54 ( 95% CI 0.23–1.28; *p* = 1.3) 3. In male T group, cancer risk: 0.35 ( 95% CI 0.13–0.96; *p* = 0.03)	General symptom: C:23 (12%) T:19 (9.9%)	Headache (C:4, T:4), Increasing heartbeat (C:2, T:2), Rash (C:4, T:4) Sweating (C:3, T:2), Increasing blood pressure (C:8, T:4), Nasal bleeding (C:2, T:3)
Seo (2014) [23]	DB, parallel	Postmenopausal women, 45–60	C:36 T:35	Korean red ginseng powder, 3 g, 12 weeks	1. Increased SOD activity 2. MDA, GPx, 8-OHdG: NS	Not reported	-
Kim (2012) [24]	DB, parallel	Healthy subjects, 20–65	C:19 T1 (low-dose): 19 T2 (high-dose):19	Korean red ginseng powder, 3 g, 6 g, 8 weeks	1. Increased SOD, GPx, catalase in T2 2. Decreased 8-epi-PGF_2_a, oxidized LDL and DNA damage in T1 and T2	Not reported	-
Kim (2011) [25]	DB, parallel	Healthy subjects, 21–61	C:27 T1 (low-dose): 27 T2 (high-dose):27	20% ethanol extract of *Panax ginseng* 1 g, 2 g, 4 weeks	1. Decreased serum ROS and MDA level in both T1 and T2 2. Increased total GSH and GSH-reductase in T2 3. TAC, catalase, SOD, GPx: NS	C:0 T1:0 T2:2 (females)	Insomnia and palpitations (T2:1) None-health related reasons (T2:1)
Kim (2013) [26]	DB, parallel	Subject with idiopathic chronic fatigue, 20–65	C:30 T1 (low-dose):30 T2 (high-dose):30	20% ethanol extract of *Panax ginseng* 1 g, 2 g, 4 weeks	1. Improved fatigue score (mental NRS and VAS): in both T groups 2. Decreased ROS and MDA in both T groups 3. Increased GSH and GSH reductase	T1:1 (female) T2:1 (male)	Non-medical reason (T1:1) Allergic response (systemic rash, pruritus) (T2:1)
Jung (2011) [27]	DB, Parallel	Healthy male subject, 19–22	C:9 T:9	Korean red ginseng extract 60 g, 11 days	1. Decreased CK and IL-6 post-uphill tread mill exercise 2. Reduced plasma glucose and insulin in OGTT	Not reported	-
Yoon (2008) [28]	DB, parallel	Healthy male subject, 19–22	C:7 (endurance training+placebo) T1:7 (endurance training+ginseng) T2:10 (only ginseng)	Korean red ginseng extract 3 g, 8 weeks	1. VO_2_max, %VO_2_/VO_2_max, Plasma BCAA among groups: NS	Not reported	-
Kulaputana (2007) [29]	DB, parallel	Physically active Thai men, 17–22	C:30 T:30	Ginseng powder, 3 g, 8 weeks	1. LT, physical performances (exercise heart rate, total exercise time, peak power output): NS 2. Oxidation rate of fat or carbohydrate: NS	None	-
Oh (2010) [30]	DB, crossover	Menopausal women, 40–60	C and T:28	Korean red ginseng powder, 3 g, 8 weeks	1. Improved FSFI in sexual arousal and GAQ	C:0 T: 2	Vaginal bleeding (T:2)
Ham (2009) [31]	DB, parallel	Patient with erectile dysfunction, 40–70	C:34 T:35	Korean red ginseng extract powder:total ginsenoside (~90%) (1:1), 0.8 g, 8 weeks	1. Improved erectile function and sexual desire in IIEF	C:5 T:8	Acute nasopharyngitis (C:3) Rhinitis (T:1) Eczema (T:1) Skin disease (T:1) Diarrhea (T:1) Anal bleeding (C:1) Voice disorders (T:1) Ophthalmalgia (T:1) Perineal pain (T:1) Chest pain (T:1) Renal stone (C:1)
Kim (2009) [32]	DB, cross-over	Women depressed sexual function 30–45	C and T:24	Korean red ginseng powder, 6 g, 6 weeks	1. FSFI (sexual function) and SF-36 (QOL): NS	No significant adverse events related to red ginseng	-
Kim (2009) [33]	DB, parallel	Patients with erectile dysfunction 33–79	C:21 T:65	Tissue-cultured mountain ginseng extract, 2 g, 8 weeks	1. Improved erectile function and overall satisfaction in IIEF	Not reported	-
de Andrade (2007) [34]	DB, parallel	Patients with erectile dysfunction, 26–70	C:30 T:30	Korean red ginseng powder, 1 g, 12 weeks	1. Improved erectile function and rigidity in IEF	C:0 T:3	Headache, insomnia
Kim (2006) [35]	DB, parallel	Patients with erectile dysfunction C: 36.1 ± 5.6 T: 43.6 ± 14.1	C:12 T:23	Tissue-cultured mountain ginseng extract, 2 g, 12 weeks	1. Improved erectile function in patients with low IEF (<17)	C:1 T:0	Minor dyspepsia (C:1)
Kim (2012) [36]	DB, parallel	Menopausal women, 45–60	C:36 T:36	Korean red ginseng powder, 3 g, 12 weeks	1. Improved Kupperman index and menopause rating scale score 2. Decreased cholesterol, LDL cholesterol and carotid intima-media thickness	Not reported	-
Kim (2009) [37]	DB, parallel	Menopausal women, 45–55	C:12 T:14	Korean red ginseng powder, 0.9 g, 8 weeks	1. Improved frequency of hot flushes	None	-
Cho (2013) [38]	DB, parallel	Non-diabetic healthy subjects with BMI ≥ 23 kg/m^2^, 20–60	C:34 T:34	Korean red ginseng powder, 6 g, 12 weeks	1. Insulin sensitivity and metabolic biomarkers: NS	C:3 T:0	Increased appetite (C:3)
Kwon (2011, 2012) [7,8]	DB, parallel	Obese women with BMI ≥ 25 kg/m^2^, 18–65	C:26 T:24	Korean red ginseng powder, 6 g, 8 weeks	1. Improved BMI and KOQOL depending on genotype 2. BMI: NS	None	-
Lee (2012) [39]	DB, parallel	Healthy subjects, 30–70	C:49 T:50	Korean red ginseng extract, 3 g, 12 weeks	1. Lowered the frequency of acute respiratory illness 2. Symptom duration and score: NS	C:7 T:11	Gastritis (T:5) Arthritis (T:2) Urticarias (C:2) Others (T:4, C:5)
Jung (2011) [40]	DB, parallel	Patients with allergic rhinitis, 19–48	C:29 T:30	Fermented red ginseng powder, 1.5 g, 4 weeks	1. TNSS score and TNSS duration score: NS 2. Improved RQOL	None	-
Han (2013) [41]	DB, crossover	Healthy male subjects, 15–37	C and T:15	Korean red ginseng powder, 4.5 g, 7 days	1. Increased total sleep time and sleep efficiency 2. Reduced total wake time	Not reported	-
Lee (2010) [42]	DB, parallel	Healthy male subjects, 19–25	C:7 T:8	Korean red ginseng powder, 4.5 g, 2 weeks	1. Total sleep, sleep latency and sleep efficiency: NS 2. Increased stage 3 sleep 3. Deceased stage 2 sleep	Not reported	-
Kitaoka (2009) [43]	DB, parallel	Healthy male subjects, 20.69 ± 0.44	C:8 T:8	Fermented red ginseng powder, 1.845 g, 8 days	1. Improved sleep efficiency in the first night without affecting sleep architecture	Not reported	-
Doosti (2014) [44]	DB, parallel	Male textile workers, 28–50	C:16 T: 6 Drug:16	*Panax ginseng* extract (G115) 200 mg, 14 days	1. Reduced noise-induced temporary threshold shift	Not reported	-
Braz (2013) [45]	DB, parallel	Patients with fibromyalgia, 27–58	C:13 T1(ginseng): 12 T2(amitriptyline):13	*Panax ginseng* extract, 100 mg, 12 weeks	1. Pain, fatigue, sleep quality and anxiety using VAS: NS 2. QOL using the FIQ: NS	Not reported	-
Lee (2012) [46]	DB, parallel	Healthy Korean subjects, 16–60	C:57 T1(low-dose:56 T2(high-dose):57	20% ethanol extract of *Panax. ginseng*, 1 g, 2 g, 4 weeks	1. Hematological and biochemical tests: NS 2. Total adverse event, symptom and sign (dyspepsia, hot flush, insomnia, constipation): NS	C:0 T1:0 T2:2 (female)	Rapid heartbeat and insomnia (T2:1) Rash and nausea (T2:1)
Park (2010) [47]	DB, parallel	Xerostomatic patients, 19–76	C:50 T:50	Korean red ginseng powder, 6 g, 8 weeks	1. Dry mouth, USFR and SSFR, symptom questionnaire: NS 2. Improved dry mouth in menopausal women (40–59 yr)	C:9 T:7	Dyspepsia (C:2,T3) Diarrhea (C:3, T:1) Itching sensation (C:2, T:1) Mild fever (C:1, T:1) Palmar sweating (C:1, T;1)-
Kang (2009) [48]	DB, parallel	Normal subjects, C: 25.6 ± 3.8 T: 27.5 ± 5.1	C:18 T:21	Korean red ginseng powder, 3 g, 3 weeks	1. Reduced SCL-90-R somatization scale	Not reported	-
Kim (2009) [49]	DB, parallel	Patients with male and female pattern alopecia	C:20 T:20	Korean red ginseng powder, 3 g, 24 weeks	1. Improved hair density and thickness	C:0 T:1	Dyspepsia (T:1)
Seo (2005) [50]	DB, parallel	Healthy male Koreans (160) and Chinese (160), 20–29	C:32 T1 (red ginseng) T2 (white ginseng):32 T3 (American ginseng 4 yrs.):32 T4 (American ginseng 6 yrs.):32	Korean red ginseng (6 yrs.), Korean white ginseng (6 yrs.), American ginseng (4 or 6 yrs.) 3 g, 4 weeks	1. No significant general symptom in Koreans 2. Increased frequency of chest discomfort in T3 and T4 Chinese group	No significant frequency of adverse events between Koreans and Chinese group	Chest discomfort in Chinese group treated American ginseng
Yang (2014) [9]	Single blind, parallel	Healthy women, 21–30	C:11 T:11	Korean red ginseng powder, 2.7 g, 2 weeks	1. Decreased urinary BPA and MDA levels 2. Alleviated menstrual irregularity, menstrual pain and constipation	None	-
Lee (2014) [51]	DB, parallel	Postmenopausal women, 50–73	C:44 T:49	Fermented red ginseng powder, 2.1 g, 2 weeks	1. Improved cognitive depression using BDI 2. Increased DHEAS, and lowered HOMAIR,	Not reported	-

APPT, activated partial thromboplastin time; AUC, area under the curve; BCAA, branched-chain amino acid; BDI, Beck Depression Inventory Questionnaire; BPA, bisphenol A; BMI, body mass index; CK, creatine kinase; CRP, high-sensitivity C-reactive protein; DB, double blind; DHEAS, dehydroepiandrosterone sulfate; DITI, Digital Infrared Thermographic Imaging; E2, estradiol; FBG, fasting blood glucose; FIQ, Fibromyalgia Impact Questionnaire; FSFI, Female Sexual Function Index; GAQ, Global Assessment Questionnaire; GH, growth hormone; GPx, glutathione peroxidase; GSH, glutathione; HbA1C, glycated hemoglobin; HOMA-IR, Homeostatic Model Assessment-Insulin Resistance; IFG, impaired fasting glucose; IIEF, International Index of Erectile Function; IL-6, interleukin 6; KOQOL, Korean version of obesity-related quality of life scale; LDL, low-density lipoprotein; LT, lactate threshold; MDA, malondialdehyde; NS, no significance between control and treatment; OGTT, oral glucose tolerance test; 8-OHdG, 8-hydroxydeoxyguanosine PT, prothrombin time; QOL, Quality of Life; ROS, reactive oxygen species; RQOL, Rhinitis Quality of Life; SCL-90, Symptom checklist-90-revised; SF-36, The 36-item Short-Form Health Survey; SOD, super oxide dismutase; SSFR, stimulated salivary flow rate; TAC, total antioxidant capacity; TNSS, total nasal symptom score; T2DM, type 2 diabetes mellitus; USFR, unstimulated salivary flow rate; USFR, unstimulated salivary flow rate; VAS, Visual Analog Scale; VO_2_max, maximal oxygen uptake.

Daily intake dosages varied based on the type of ginseng. The *Panax ginseng* extract (G115) intake was 100–400 mg, while the 20% ethanol ginseng extract intake was 1–2 g. The dosage for Korean red ginseng powder was 0.9–6 g, with the most common dosage being 3 g in 8 studies. Korean red ginseng extract intake was between 200 mg and a maximum of 60 g per day. Fermented red ginseng powder intake was 1.5–2.7 g, and cultured ginseng extract intake was 2 g. One study administrated 3 g of *Panax ginseng* and *Panax quinquefolium* each to compare the adverse events between Korean and Chinese participants.

The duration of ginseng intake was 7–11 days for the short-term studies (4 studies), and the maximum duration was 3 years. About 50% of the studies had intakes of 8 or 12 weeks. There were 13 studies with an 8-week intake, 9 studies with a 12-week intake, 7 studies with a 4-week intake, 6 studies with a 2-week intake, 2 studies with a 6-week intake, and one study each with a 24-week, 3-week, and 3-year intake.

### 3.4. Efficacy

According to the primary endpoint results, 29 studies showed statistical significance between the placebo groups and ginseng groups, while 15 studies did not show any effects. Two studies from the 15 studies showed no effect on the biomarkers but did show an improved quality of life.

Study results showed that Korean red ginseng extract inhibited platelet aggregation in healthy subjects [13]. There was no significant thermal effect from ginseng caused by improved blood circulation in females with a low Female Sexual Function Index (FSFI) value [12] and in healthy subjects [10]. There was also no effect on blood pressure in patients with metabolic syndrome [11].

Korean red ginseng powder reduced insulin and C-peptide during oral glucose tolerance tests (OGTT) in patients with mild diabetes [14]. Fermented red ginseng also decreased blood glucose, glycated hemoglobin (HbA1C) and Homeostatic Model Assessment-Insulin Resistance (HOMA-IR) levels during OGTT in healthy or postmenopausal females [15,16], but Korean red ginseng extract had no effect on glucose metabolism in overweight subjects or newly diagnosed type 2 diabetes mellitus (T2DM) patients [17]. G115 had no effect on blood glucose-related markers in healthy subjects [18]. 

Korean red ginseng extract improved working memory in the psychomotor function study [21], but there was no effect from Korean red ginseng powder on the neurocognitive function tests in healthy subjects [19]. *Panax ginseng* extract G115 showed no effect on working memory [20]. 

There was a decrease in cancer risk in chronic atrophic gastritis patients with 3 years of intervention with the Korean red ginseng extract in an 8-year follow-up study [22].Korean red ginseng powder and the 20% ethanol ginseng extract showed antioxidative effects in postmenopausal females or healthy subjects [23,24,25].

Twenty percent ethanol ginseng extract improved fatigue scores and showed antioxidative effects in chronic fatigue patients [26]. Korean red ginseng extract decreased creatine kinase (CK) and interleukin 6 (IL-6) levels after uphill tread mill exercise [27]. However, Korean red ginseng extract and ginseng powder did not affect maximal oxygen uptake (VO_2_max) or physical performance in healthy subjects [28,29].

One study showed that Korean red ginseng powder improved the FSFI values for sexual arousal and the Global Assessment Questionnaire (GAQ) values in menopausal females [30], but another study showed no effect on the FSFI values in women with sexual dysfunction [32]. A group of studies showed Korean red ginseng powder and cultured ginseng extract improved erectile dysfunction [31,33,34,35]. 

Korean red ginseng powder improved menopausal symptoms such as hot flushes and decreased blood cholesterol and LDL-cholesterol levels in menopausal females [36,37].

Korean red ginseng powder had no effect on metabolic biomarkers such as insulin sensitivity in non-diabetic healthy subjects with BMIs greater than 23 kg/m^2^ [38], and did not improve BMIs in a study on obese female subjects (BMI ≥ 25 kg/m^2^) [7]. However, it showed a genotype-dependent improvement in BMI and quality of life [8]. Korean red ginseng extract reduced the frequency of acute respiratory illnesses but not the symptom durations [39]. Fermented red ginseng improved the quality of life although it did not show any improvement related to allergic rhinitis symptoms [40].

Korean red ginseng powder and fermented red ginseng improved the effective sleep time and sleep efficiency [41,42,43]. Korean red ginseng powder improved somatic symptoms [48], hair thickness and hair density [49]. It did not improve dry mouth symptoms in xerostomatic patients, but an intergroup analysis showed improvement in dry mouth symptoms in menopausal females [47]. Korean red ginseng powder also reduced urinary BPA and MDA levels in young females and reduced the severity of gynecological complaints [9]. Fermented red ginseng improved cognitive depression in postmenopausal females [51]. *Panax ginseng* extract (G115) reduced noise-induced temporary threshold shifts in textile workers [44], but had no effect on pain and fatigue in patients with fibromyalgia [45]*.*

### 3.5. Safety

Adverse events from the selected studies are summarized in Table 2. From the 44 selected studies, 23 studies did not report on adverse events, 5 studies reported no adverse events, and 16 studies reported adverse events. Two studies in particular focused on safety. One study compared the adverse events of red ginseng and white ginseng prepared from *Panax ginseng* with those of American ginseng prepared from *Panax quinquefolium* for a 4-week intervention using 3 g in healthy Korean and Chinese subjects [50]. In that study, there was no difference in the frequencies of the adverse events in the Korean subjects among the three ginseng types. However, a significant increase of chest discomfort was reported after the intake of American ginseng in Chinese subjects. In the other safety study with a 4-week intervention using 1 or 2 g of 20% ethanol ginseng extract of *Panax ginseng*, the four most frequently reported adverse events were dyspepsia, hot flushes, insomnia and constipation. However, there were no differences compared with the placebo group and no changes in hematologic and biochemical markers [46].

In the study with a long-term intervention of 3 years in chronic atrophic gastritis patients, the adverse events were headaches, increased heartbeat, rashes, sweating, raised blood pressure and nasal bleeding. However, there was no significant difference in frequency between the placebo and ginseng groups, which were 12% and 9.9%, respectively [22].

Adverse events occurring during the clinical trials were reported to have no relation with the test sample; allergic reactions such as cold allergy were reported in the ginseng group, but the author stated that it was not related to the ginseng intervention [13]. Adverse events such as voice disorder, ophthalmalgia, perinea pain and renal stones in the erectile dysfunction study were shown to have no relation with the test sample [31]. In a 12-week intervention study with healthy subjects, there were no statistical differences between the placebo and ginseng groups for adverse events, and specific events were not observed (*p* = 0.378) [39]. The observed adverse events of gastritis, arthritis and urticaria during the study period had no causal relationship to the ginseng intake [39]. Vaginal bleeding was reported in the study on sexual dysfunction in menopausal females [30]. One case of hypoglycemia was reported in the glucose metabolism study with healthy subjects [15].

**Table 2:** Frequency of adverse events in this review.

Adverse Event	Placebo Control (*n* = 1381)	P. Ginseng (*n* = 1711)
Dyspepsia	13 (9.6 ^a^ , 0.9 ^b^)	18 (9.4 ^a^, 1.1 ^b^)
Hot flash	19 (14.1 , 1.4)	34 (17.8, 2.0)
Insomnia	9 (6.7 , 0.7)	20 (10.5, 1.2)
Constipation	6 (4.4 , 0.4)	10 (5.2, 0.6)
Low energy	1 (0.7 , 0.1)	4 (2.1, 0.2)
Headache	10 (7.4 , 0.7)	11 (5.8, 0.6)
Skin disorders	6 (4.4 , 0.4)	16 (8.4, 0.9)
Dizziness	7 (5.2 , 0.5)	6 (3.1, 0.4)
Nausea	1 (0.7 , 0.1)	2 (1.0, 0.1)
Diarrhea	10 (7.4 , 0.7)	12 (6.3, 0.7)
Abdominal pain	0	2 (1.0, 0.1)
Nasal Bleeding	5 (3.7 , 0.4)	10 (5.2, 0.6)
Rapid heartbeat	2 (1.5 , 0.1)	5 (2.6, 0.3)
Anorexia	6 (4.4 , 0.4)	3 (1.6, 0.2)
Increased appetite	3 (2.2 , 0.2)	0
Dried mouth	13 (9.6 , 0.9)	12 (6.3, 0.7)
Chest discomfort	8 (5.9 , 0.6)	9 (4.7, 0.5)
Eruption on the tongue	1 (0.7, 0.1)	0
Allergy (cold allergy, systemic rash)	0	3 (1.6, 0.2)
Common cold	0	2 (1.0, 0.1)
Itching sensation	2 (1.5, 0.1)	2 (1.0, 0.1)
Mild fever	1 (0.7, 0.1)	1 (0.5, 0.1)
Sweating	4 (3.0, 0.3)	3 (1.6, 0.2)
Increasing blood pressure	8 (5.9, 0.6)	4 (2.1, 0.2)
Vaginal bleeding	0	2 (1.0, 0.1)
Total events	135 (9.8 ^b^)	191 (11.2 ^b^)

^a^, adverse events/total adverse events of each group × 100; ^b^, adverse events/total participants of each group × 100.

Adverse events reported in the RCTs were described as general symptoms which were also observed in the placebo group. General symptoms for adverse events such as hot flushes, insomnia, dyspepsia, skin disorders, dried mouth, diarrhea, headaches, chest discomfort, constipation, nasal bleeding, dizziness, rapid heartbeat and anorexia were reported in both ginseng and placebo groups.

Overall, there was no statistical significance between the ginseng and placebo groups on the frequency or symptoms of adverse events, and no serious or severe adverse events were reported. The symptoms were mild and temporary, which ceased when ginseng administration was discontinued.

## 4. Discussion

*Panax ginseng* C.A. Meyer not only has been used as a traditional tonic and medicine for thousands of years in Asia, but also as a dietary supplement and complementary medicine and for adjuvant therapeutics worldwide. It is necessary to provide information on its safety along with its clinical efficacy to consumers as the demand for ginseng increases. 

From clinical trials on the efficacy and safety of ginseng, randomized controlled clinical trials over the past 10 years were searched for in online databases and the reported adverse events were investigated. Because the use of other herbal medicines or drugs combined with ginseng could cause interactive adverse events, we selected 45 articles that used ginseng as a monopreparation as well as those meeting other criteria. However, 15 studies out of the 44 studies were shown to be not significant. For example, in five studies on blood glucose regulation, three studies showed an effect on glucose metabolism in impaired fasting glucose or impaired glucose tolerance, in menopausal female or healthy subjects [14,15,16]. The test samples, dosages and durations also varied in these studies. These results indicate that subject selection, dosage, duration and biomarkers in clinical trials are crucial for obtaining positive results. In addition, clearly stating the production process and the content of the samples used can support the reliability of the study. Although many systematic reviews on clinical trials with ginseng have been published recently, many of them have a high risk of bias due to the small number of participants [5,6,52,53]. 

The short-term interventions were between 7–11 days while one long-term intervention was three years. Fifty percent of the studies were between 8–12 weeks, and the rest of studies were less than six weeks. Therefore, moving forward, long-term studies of more than 12 weeks should be done on the efficacy and safety of ginseng.

Among the reviewed studies that used ginseng as a monopreparation and in which no other drugs were taken, five studies reported no adverse events, and 16 studies reported adverse events. The reported adverse events were mainly general symptoms that were observed in both the ginseng and placebo groups such as dyspepsia, hot flushes and insomnia. These adverse events were mild and temporary. There were no significant differences in the frequency and symptoms of adverse events between the placebo and ginseng groups, indicating that ginseng intake is safe generally. However, 23 studies did not report on adverse events. Because not many studies are discussing adverse events sufficiently, the evaluation of safety of *Panax ginseng* in this review could be limited. To fully understand the adverse events of ginseng, studies should report detailed adverse events during the trials.

In the case of adverse events from ginseng, interaction with warfarin has been reported in some case reports [54,55], but it was also reported that there was no interaction with warfarin in healthy subjects [56] and in patients receiving warfarin therapy [57,58]. Although many case reports have reported on ginseng and drug interaction, the evidence is insufficient because the studies lack clear information on the samples which were combined with other herbs or on the condition of the participants. Because many people take drugs to maintain their health and expect supplementary effects from ginseng, it will be necessary to provide clear information to ginseng consumers through studies on drug interaction, on the activities of drug metabolizing enzymes, and on drug transporters and pharmacokinetics.

Furthermore, studies on the efficacy and safety of ginseng with higher standards should be conducted to help consumers maintain health and combat diseases with ginseng as a dietary supplement or complimentary medicine.

## 5. Conclusions

*Panax ginseng* as a monopreparation showed a safe profile with no significant differences between the placebo and ginseng groups in terms of the frequency and symptoms of adverse events in RCT studies with a small number of subjects with various conditions for each patient. However, more studies with higher standards and larger-scaled clinical trials on the efficacy and safety of ginseng are necessary to provide more definite information about ginseng as a functional food or complementary medicine to the consumers.
